# Insights into the structures and electronic properties of Cu_n+1_^*μ*^ and Cu_n_S^*μ*^ (n = 1–12; *μ* = 0, ±1) clusters

**DOI:** 10.1038/s41598-017-01444-6

**Published:** 2017-05-02

**Authors:** Cheng-Gang Li, Zi-Gang Shen, Yan-Fei Hu, Ya-Nan Tang, Wei-Guang Chen, Bao-Zeng Ren

**Affiliations:** 1 College of Physics and Electronic Engineering, Quantum Materials Research Center, Zhengzhou Normal University, Zhengzhou, 450044 China; 2 School of Chemical Engineering and Energy, Zhengzhou University, Zhengzhou, 450001 China; 3 School of Physics and Electronic Engineering, Sichuan University of Science & Engineering, Zigong, 643000 China

## Abstract

The stability and reactivity of clusters are closely related to their valence electronic configuration. Doping is a most efficient method to modify the electronic configuration and properties of a cluster. Considering that Cu and S posses one and six valence electrons, respectively, the S doped Cu clusters with even number of valence electrons are expected to be more stable than those with odd number of electrons. By using the swarm intelligence based CALYPSO method on crystal structural prediction, we have explored the structures of neutral and charged Cu_n+1_ and Cu_n_S (n = 1–12) clusters. The electronic properties of the lowest energy structures have been investigated systemically by first-principles calculations with density functional theory. The results showed that the clusters with a valence count of 2, 8 and 12 appear to be magic numbers with enhanced stability. In addition, several geometry-related-properties have been discussed and compared with those results available in the literature.

## Introduction

Because of the peculiar electronic structure (3*s* ^2^3*p* ^6^3*d* ^10^4*s* ^1^) of the Cu atom with one *s* electron outside a closed *d* shell, the small energy difference between the atomic *s* and *d* levels leads to strong hybridization effects, which may play an important role in determining the structures and electronic properties of copper clusters. In recent years, the structure and properties of copper clusters have received considerable attention from both the experimental and theoretical points of view^1–21^. Many experimental data such as the bond length, frequency, binding energy, vertical ionization potentials (VIP) and vertical electron affinities (VEA), adiabatic detachment energy (ADE) and vertical detachment energy (VDE) are reported for pure neutral and charged copper clusters^1–13^. With the development of rigorous calculation methods, numerous theoretical works have been performed by different programs, such as SIESTA, deMon-KS, ALLCHEM, Gaussian etc.^14–21^. For example, Jaque and Labbe^14^ studied the molecular structure, binding energy, electronic properties and reactivity descriptors using the GAUSSIAN09 package for nine neutral copper clusters. Ramirez *et al*.^15^ presented the minimum energy structures of Cu_n_^*μ*^ (*μ* =±1, 0, 2; n = 3–13) clusters through a joint gradient embedded genetic algorithm (GEGA) technique. Jug *et al*.^16^ investigated the structure and stability of neutral and ionic Cu_n_ clusters (n ≤ 10) using the DFT program ALLCHEM, calculations on the LDA level are proved to be a suitable mean for the determination of structure and frequency. GGA corrections are needed for correct prediction of the relative stability of isomers and the global minimum. Density functional theory calculations of copper cluster Cu_n_ (n = 2–10) are analyzed with respect to their molecular orbitals. Results showed that shell type orbitals of *s*, *p* and *d* character govern the electronic structure growth and have an influence on the geometric structure^17^. Using the demon-KS package, Calaminici *et al*.^18^ reported the LCGTO-DFT local and GGA first principles all-electron calculations for the structural and spectroscopic properties of neutral and charged Cu_n_ (n ≤ 5) clusters. On the basis of the first-principles code SIESTA, Fernandez *et al*.^19^ preformed a systematic study of the electronic properties and geometric structure of copper clusters Cu_n_^*μ*^ (n ≤ 13 and n = 20; *μ* = 0, ±1). The maximum size of planar clusters are determined at n = (5, 6, 4) for anionic, neutral and cationic copper, respectively. Moreover, the trends of the cohesive energy, ionization potentials, electron affinities, and highest occupied–lowest unoccupied molecular orbital (HOMO-LUMO) gap are studied in detail as the cluster size increase and charge state changes.

As we know that the structures and properties of clusters are very sensitive to the number of atoms, which can change dramatically with the addition or substitution of one atom. The introduction of a doped atom in copper clusters, such as a sulfur atom in our work, can undoubtedly change the clusters’ structure, which in further alters their physical and chemical properties significantly. Copper sulfides have attracted sustained research interests over the past decades due to their wide applications in solar cell devices, nonlinear optical materials, lithium ion batteries, nanometer-scale switches and gas sensors^22–25^. For example, the electronic structures of Cu_2_S cluster were calculated in the local density functional approximation^26^. Their theoretical results suggested that the structures closely resemble an S^−^ bridging a Cu_2_^+^ as shown by the geometries and charge distributions. The potential energy surface minima of neutral copper sulfide clusters have been explored using the Coalescence Kick global optimization method and combined with a DFT approach^27^. The calculated HOMO-LUMO gap (1.3 to 3.3 eV) showed that the (CuS)_n_ clusters can be considered as a suitable candidate for renewable energy sources. At last, Scott *et al*.’s results confirmed that Cu-S-Cu units can be found in an active site of some metalloproteinase like cytochrome *c*-oxidase^28^. The short Cu-Cu distance and small Cu-S-Cu bond angle play an essential role in the electron transport made by this protein.

It is well known that the Jellium model plays a significant role in the realm of cluster. It incorporates a positively charged (spherical or non-spherical) background potential, which results in discrete energy levels of the delocalized (“metallic”) electrons corresponding to angular momentum shells (in the spherical case the shells are labeled as: 1S^2^ 1P^6^ 1D^10^ 2S^2^ 1F^14^, 2P^6^, 1G^18^…)^29^. There are certain valence electronic configurations (2, 8, 18, 20, 40, 58…) know as magic numbers, that exhibit increased stability relative to their neighboring configurations. So, these clusters are generally resistant to reactivity with small clusters^30–34^. For example, Jaque *et al*.^14^ studied the molecular structures, binding energy, electronic properties and reactivity descriptors for the neutral copper cluster, and found that the large HOMO-LUMO energies gap, minimum polarizability and maximum hardness corresponding with the 2 and 8 valence electrons are operative for characterizing and rationalizing the electronic properties of copper clusters. However, other groups found that some clusters with the non-magic number total electrons can also exhibit an increased stability^35, 36^. For example, Rebere *et al*.^36^ reported that Ag_15_^+^, Ag_14_ and Ag_13_^−^ clusters with 14 valence electrons are resistant to reactivity with oxygen, even though they do not have a magic number of electrons. This simulated further and more detailed investigations on the magic numbers.

Although the structural and electronic properties of copper and sulfur doped copper clusters have been investigated by both experiment and theory, some important question arises: (i) For different copper clusters with the small energy difference between the atomic *s* and *d* levels, how does hybridization effect from different molecular orbitals? (ii) How does the structural vary as a consequence in anionic and cationic Cu_n_S clusters? Furthermore, are their structures and properties greatly distinct from the neutral Cu_n_S clusters? (iii) In view of the fact that the electronic structure of Cu and Ag featuring a closed *d* shell and a singly occupied *s* shell (3*d* ^10^4*s* ^1^ and 4*d* ^10^5*s* ^1^), whether the Jellium model is in accordance with the pure copper clusters? With a doped sulfur, how does the magic number vary? (iv) For the charged Cu_n_S clusters, how does HOMO-LUMO gap change? Hence it is necessary to carry out a systematic investigation to reveal the magic number, structures and electronic properties of copper and sulfur doped copper clusters.

With this purpose in mind, we perform a systematic, in–depth study of the geometric structures and electronic properties for neutral Cu_n+1_ and Cu_n_S (n = 1–12) clusters and their ions based on particle swarm optimization algorithm and density function theory. Thus, we are able to advance a fundamental understanding of the ground state geometric configurations. In addition, the magic numbers, HOMO-LUMO gaps, density of states (DOS), adaptive natural density partitioning (AdNDP), electron localization function (ELF) and Mayer bond order are also analyzed. Subsequently, the calculated values are compared with available experimental and theoretical data.

## Results and Discussions

### Structural properties

**Figure 1:**
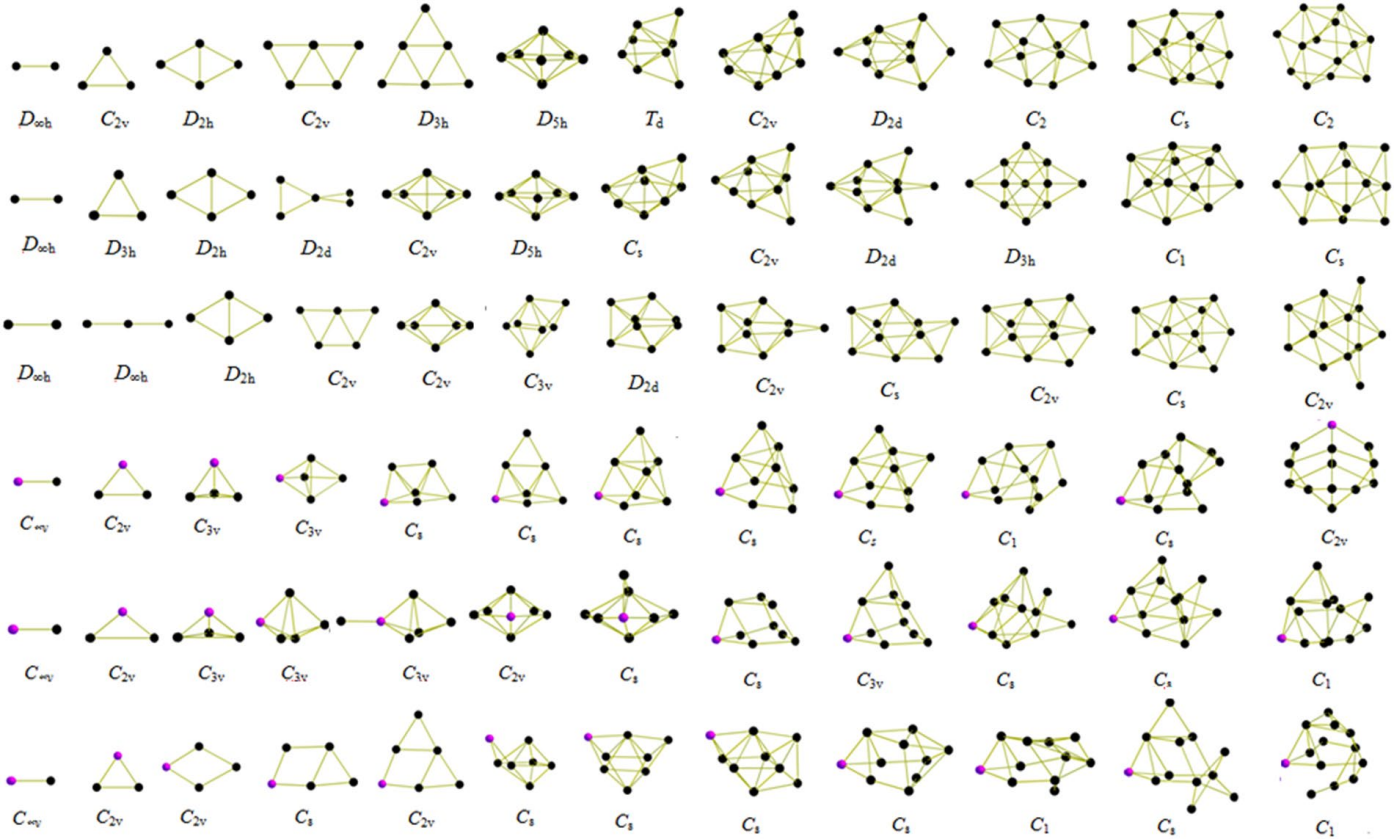
The lowest energy structures of neutral, cationic and anionic Cu_n+1_ and Cu_n_S (n = 1–12) clusters. The purple and black balls represent sulfur and copper atoms, respectively.

We performed unbiased searches for the global minima structures of neutral and charged Cu_n+1_ and Cu_n_S (n = 1–12) clusters. Previously reported structures are successfully reproduced with CALYPSO method from experimentally and theoretically. The global minimum structures for each size are displayed in Fig. 1. The corresponding electronic states, symmetries, total energies are also determined and listed in Table S1 in the Supporting Information (S1).

As depicted in Fig. 1, it is clear that the charges have important influence on the structures of pure and doped clusters. We found nearly the same structure only in the cases of n = 1, 3 for Cu_n+1_ and n = 1, 2 for Cu_n_S, respectively, while the remaining clusters contain different structures. For example, both Cu_3_^+^ and Cu_3_ have a triangular structure, while Cu_3_^−^ is composed of a linear structure. The same trend can be also observed for Cu_3_S^+/0/−^ cluster, namely, Cu_3_S^+^ and Cu_3_S possess the same triangular structure, whereas Cu_3_S^−^ has a rhombus structure. In addition, the transition from two-dimensional (2*D*) to three-dimensional (3*D*) configurations is closely related to their charge state. For non doped-cluster, the 2*D*-3*D* structure transition occurs at n = 4–5 for Cu_n_^+^, n = 6–7 for Cu_n_, and n = 5–6 for Cu_n_^−^; while for S doped ones, n = 2–3 for Cu_n_S^+^, n = 2–3 for Cu_n_S, and n = 5–6 for Cu_n_S^−^, respectively. For neutral copper cluster, two growth patterns can be obtained here. One is to remain essentially two dimensional and grow by forming successive triangle faces for n = 2–6. From n > 7, the cluster forms a pentagon bipyramid structure with the two capping atoms forming a bond. And, it becomes more closely packed with the increasing atomic number of clusters. For charged copper cluster, the ground state structures exhibit a layer-like 3*D* configuration from n = 5 to 6, which display a preference for layered and pyramidal geometries. In addition, our structures for Cu_2_^+/−^ (${D}_{\infty h}$, ${D}_{\infty h}$), Cu_3_^+/−^ (*D*_3h_, ${D}_{\infty h}$), Cu_4_^+/−^ (*D*_2h_, *D*_2h_), Cu_5_^+/−^ (*D*_2d_, *C*_2v_), Cu_6_^+/−^ (*C*_2v_, *C*_2v_), Cu_7_^+/−^ (*D*_5h_, *C*_3v_), Cu_8_^+/−^ (*C*_s_, *D*_2d_), Cu_9_^+/−^ (*C*_2v_, *C*_2v_), Cu_10_^+/−^ (*D*_2d_, *C*_s_), Cu_11_^+/−^ (*D*_3h_, *C*_2v_), Cu_12_^+/−^ (*C*_1_, *C*_s_) and Cu_13_^+/−^ (*C*_s_, *C*_2v_) are consistent with previous computational values of reference^20^. Nevertheless, it should be mentioned that a new lowest energy structure of Cu_9_ cluster is identified, which is different from previous studies^14–16^. In present work, a *C*_2v_ symmetry Cu_9_ cluster is found as the lowest energy structure; however, different isomer with *C*_s_ symmetry are reported by Jug (ALLCHEM), Jaque (B3PW91/LANL2DZ) and Ramirez *et al*. (BLYP/6-311+G(d)). The different lowest energy structures may be due to the fact that the different functional and basis sets may reverse the order of clusters in some cases. So, in order to confirm the lowest energy structure of Cu_9_ cluster, the same functional and basis sets (B3PW91/LANL2DZ) are performed based on the DFT calculations. Results showed that the Cu_9_ structure *C*_s_, reported in ref. 14 as a minimum, in our calculation is found to be the second one, at a relative energy of 0.02 eV above our minimum. In addition, it is also noted that the Gibbs free energy of Jaque *et al*.’s results is 6.3 × 10^3^ J/mol higher than our calculated work. Above analysis indicated that that our results are energetically lower and structurally more stable than Jaque *et al*.’s results.

For the neutral and charged Cu_n_S clusters, different types of clusters possess the same growth pattern with rare exceptions. Practically speaking, the doped clusters are formed by adding a copper atom into the smaller sized Cu_n−1_S clusters. Any additional S atom is just one more vertex to surround the Cu atom, and finally the cluster forms a triangular prism. In conclusion, the growth pattern of Cu_n_S^+/0/−^ clusters are quite different from Cu_n_^+/0/−^ clusters. One Cu atom directly added on the Cu_n−1_S clusters are dominant growth pattern for Cu_n_S^+/0/−^ clusters.

To check the accuracy of the lowest energy structures, several available experimental and theoretical results are calculated and compared. For Cu_n_ clusters, the evolution of VIP, VEA and static mean polarizablity (*α*) values is plotted in Figure S1, together with available experimental and calculated results^3–5, 13, 16^. For Cu_n_^−^ clusters, the calculated ADE and VDE are listed in Table 1, which also includes the available experimental values taken from ref. 1. For Cu_2_S cluster, the calculated bond length (Cu-S, Cu-Cu) and bond angle (CuSCu) (2.14 Å, 2.67 Å and 77.5°) are close to the Boris and Mahe *et. al’s*. work (2.11 Å, 2.63 Å and 76.7°)^37, 38^. In addition, we found that our calculated results about the bond length and angles, dissociation energies and frequency are also in excellent agree with the experimental and theoretical results for Cu_n_S clusters^26, 27, 37, 38^. In conclusion, we can see that our calculated values are in good agreement with the experimental and theoretical results. Thus, we believe that the choice of functional and basis sets could be reasonably good to describe the present systems.

**Table 1:** The calculated ADE and VDE for Cu_n_^−^ clusters together with the available experimental data.

Cluster	Adiabatic electron affinity (eV)		Vertical detachment energy (eV)	
Cu_n_^−^	Cal	Exp^[1]^	Cal	Exp^[1]^
2	1.15	0.84 ± 0.01	1.15	0.89 ± 0.01
3	2.26	2.30–2.50	2.43	2.35–2.55
4	1.82	1.40 ± 0.05	1.82	1.45 ± 0.02
5	2.17	1.92 ± 0.05	2.17	1.97 ± 0.02
6	1.80	1.92 ± 0.05	2.29	1.97 ± 0.02
7	2.34	2.10 ± 0.05	2.13	2.15 ± 0.02
8	1.74	1.53 ± 0.05	1.77	1.58 ± 0.02
9	2.57	2.30–2.60	2.87	2.35–2.65
10	2.00	1.99 ± 0.05	2.02	2.04 ± 0.02

### Stabilities and electronic properties

To analyze the relative stabilities of the lowest energy structures of Cu_n+1_^+/0/−^ and Cu_n_S^+/0/−^ (n = 1–12) clusters, the averaged binding energies *E*_b_(n) and second-order difference of energies ∆_2_ *E*(n) have been derived. *E*_b_(n) and ∆_2_ *E*(n) of each complex for Cu_n_S^+/0/−^ clusters are calculated as follows:

1 $$\begin{array}{ccc}{E}_{b}(n) & = & [nE({\rm{Cu}})+E({{\rm{S}}}^{-/0/+})-E({{\rm{Cu}}}_{n}{{\rm{S}}}^{-/0/+})]/({\rm{n}}+1)\\ {{\rm{\Delta }}}_{2}E({\rm{n}}) & = & E({{\rm{Cu}}}_{n+1}{{\rm{S}}}^{-/0/+})+E({{\rm{Cu}}}_{n-1}{{\rm{S}}}^{-/0/+})-2E({{\rm{Cu}}}_{n}{{\rm{S}}}^{-/0/+})\end{array}$$

For Cu_n_^+/0/−^ clusters, *E*_b_(n + 1) and ∆_2_ *E*(n + 1) are defined as:

2 $$\begin{array}{rcl}{E}_{b}(n+1) & = & [nE({\rm{Cu}})+E({{\rm{Cu}}}^{-/0/+})-E({{\rm{Cu}}}_{n+1}^{-/0/+})]/({\rm{n}}+1)\\ {{\rm{\Delta }}}_{2}E({\rm{n}}+1) & = & E({{\rm{Cu}}}_{n+2}^{-/0/+})+E({{\rm{Cu}}}_{n}^{-/0/+})-2E({{\rm{Cu}}}_{n+1}^{-/0/+})\end{array}$$

**Figure 2:**
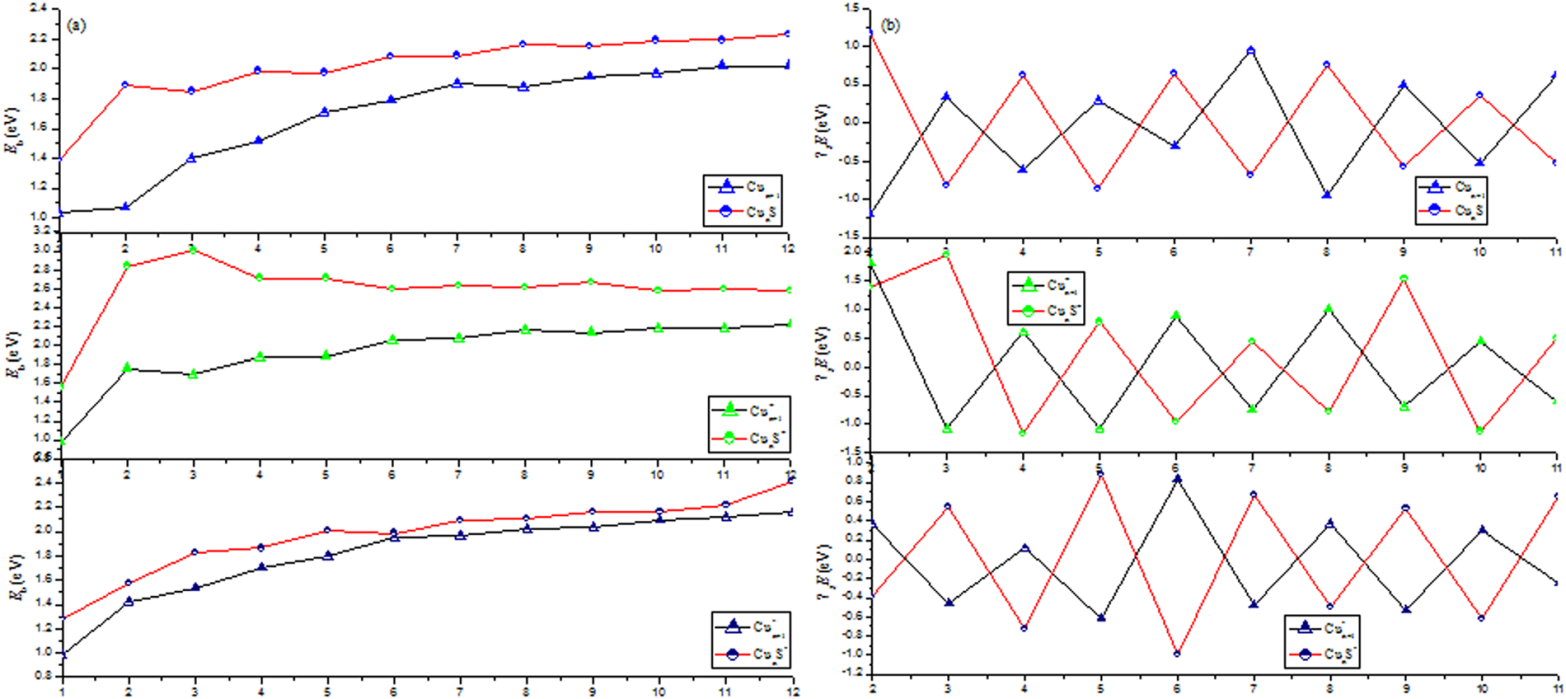
Calculated average binding energies and second-order energy differences of neutral and charged Cu_n+1_ and Cu_n_S (n = 1–12) clusters.

The calculated *E*_*b*_(*n*) and ${{\rm{\Delta }}}_{2}E(n)$ as a function of the number *n* of copper atoms are plotted in Fig. 2. As by comparing *E*_*b*_(n) for both Cu_n_S^+/0/−^ and Cu_n_^+/0/−^ clusters, the primary features are concluded: (i) The values of ${E}_{b}({{\rm{Cu}}}_{n}{{\rm{S}}}^{-/0/+})$ clusters are higher than those of ${E}_{b}({{\rm{Cu}}}_{n}^{-/0/+})$ clusters, implying that the doped S atom can enhance the stability of host Cu_n_^+/0/−^ clusters. (ii) For Cu_*n*_S clusters, there are six visible peaks in the curves at n = 2, 4, 6, 8, 10 and 12, indicating that Cu_2, 4, 6, 8, 10, 12_S are relatively more stable than its neighboring clusters. For Cu_*n*_S^−/+^ clusters, Cu_3_S^−^, Cu_5_S^−^, Cu_7_S^−^, Cu_9_S^−^ and Cu_3_S^+^ have a higher relative stability than their neighbors, respectively. (iii) For Cu_n+1_^+/0/−^ clusters, *E*_b_(n) have a monotonically increasing with an increase of copper atoms, revealing an enhanced effect on the stabilities of the Cu_n+1_^+/0/−^ clusters with the cluster size increasing. In addition, the charged clusters have larger values for the binding energy than neutral ones, while ionic clusters possessing the largest values lie in between them. Another important parameter to evaluate the relative stabilities is the size-dependent second energy difference, the values of neutral and charged clusters exhibit obvious odd-even alternations as shown in Fig. 2. In which the charged and neutral clusters are more stable with even and odd cluster size *n* for non-doped Cu_n+1_^+/0/−^, respectively. Whereas the charged and neutral clusters are more stable with odd and even cluster size *n* for doped Cu_n_S^+/0/−^, respectively. The above analysis can be explained by this can be attributed to the presence or absence of unpaired electrons of the complex. In addition, we can easily point to some conspicuous peaks: n = 2, 7, 6 for Cu_n+1_^+/0/−^ clusters as well as n = 3, 2, 5 for Cu_n_S^+/0/−^ clusters. It suggests that these clusters are more stable compared with other clusters.

**Figure 3:**
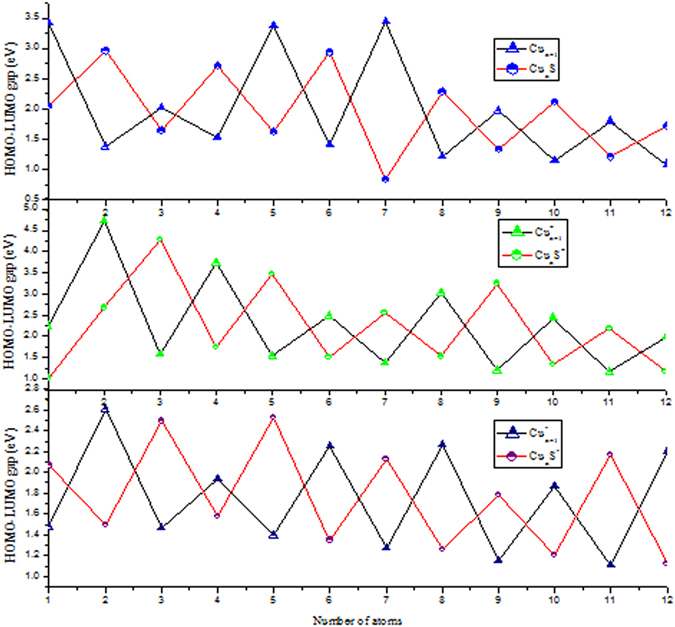
The HOMO-LUMO energy gaps for the lowest energy structures of neutral and charged Cu_n+1_ and Cu_n_S (n = 1–12) clusters.

The HOMO-LUMO energy gaps (*E*_gap_) have been proved to be a powerful tool to represent the ability of the molecule to participate in the chemical reaction in some degree. Larger values of values indicate stronger chemical stability. The calculated of HOMO-LUMO energy gaps are shown in Fig. 3 for the lowest energy structures of Cu_n+1_^+/0/−^ and Cu_n_S^+/0/−^ (n = 1–12) clusters. It is interesting to notice that Cu_n+1_ with even number of electrons are more stable than other clusters with odd number of electrons. Contrary, for Cu_n+1_^+^ and Cu_n+1_^−^ clusters, odd-even alteration behaviors of *E*_gap_ curve present the opposite trend compared to the corresponding neutral clusters. This can be explained that even numbers electrons are always exist in pairs and can lead to a closed shell electronic structure. We also observe that *E*_gap_ of cationic copper clusters are larger than their anionic counterparts, indicating that Cu_n+1_^+^ clusters are less stable, which is in excellent agreement with the results of averaged binding energies shown in the Fig. 2. As for Cu_n+1_^−^ clusters, four local maxima of *E*_gap_ are found at n = 2, 6, 8 and 12, respectively, suggesting that Cu_3, 7, 9, 13_^−^ clusters are more stable than their neighbors. The local maxima of the *E*_gap_ for Cu_n+1_ and Cu_n+1_^+^ clusters indicate that neutral Cu_2, 6, 8_ and cationic Cu_3, 5, 9_^+^ clusters possess enhanced relative stability. Similarly, for Cu_n_S^+/−^ clusters, except Cu_2_S^+^, the results of *E*_gap_ present the same odd-even oscillation. Moreover, the *E*_gap_ of Cu_n_S^−^ are always lower than their cationic counterparts with the exception of CuS^+^. Those results indicate that cationic clusters are more stable than corresponding anionic clusters. In the case of Cu_n_S^+/0/−^ clusters, some local maxima *E*_gap_ values are observed at Cu_2_S, Cu_4_S, Cu_6_S for neutral clusters, Cu_3_S^+^, Cu_5_S^+^, Cu_9_S^+^ for cationic clusters as well as Cu_3_S^−^, Cu_5_S^−^, Cu_11_S^−^ for anionic clusters. Combining the above analysis on *E*_b_(n), ∆_2_ *E*(n) and *E*_gap_ values of Cu_n+1_^+/0/−^ and Cu_n_S^+/0/−^ clusters, we can infer that Cu_8_, Cu_3_^+^, Cu_7_^−^, Cu_2_S, Cu_3_S^+^ and Cu_5_S^−^ clusters exert a remarkable chemical stability. At the same time, we checked the valence electrons of above stable clusters, the number is 2 and 8 for Cu_3_^+^ and Cu_8_, Cu_7_^−^, Cu_2_S, Cu_3_S^+^ respectively, which are magic numbers according to Jellium model. However, our theoretical predictions point out that the clusters of Cu_5_S^−^ is particularly stable despite the cluster has 12 valence electrons and do not conform to the predictions of the Jellium model. Finally, for Cu_n_S^+/0/−^ clusters, the calculated HOMO-LUMO gaps range from 1.01–4.29 eV, 0.8–2.96 eV, 1.26–2.53 eV, respectively. These are desirable band gaps of semiconductor nanomaterials, from an application point of view, it suitable for application in photocatalysis field (especially for Cu_n_S^+^ clusters). We expect that studies of the neutral and charged sulfur-copper clusters could provide favorable information in the searching functional design and application of the renewable energy sources.

**Figure 4:**
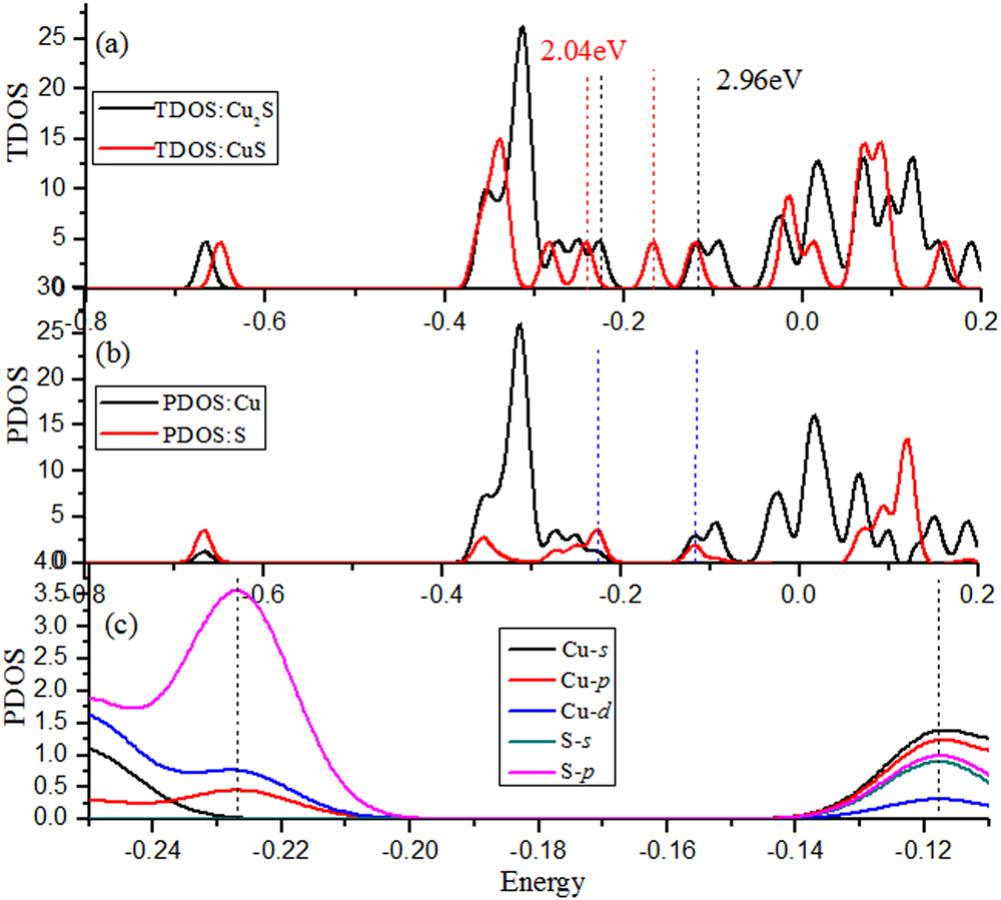
(**a**) Total densities of states for CuS and Cu_2_S clusters. (**b**) Partial densities of states of Cu_2_S clusters. (**c**) Partial densities of states for copper atom in Cu_2_S clusters. (full width at half maximum (FWHM) = 0.02 a.u.) The dashed line indicates the HOMO and LUMO energy.

To understand the nature of the chemical bonding and the formation mechanism of these various Cu-S compounds, the total density of states (TDOS) and partial density of states (PDOS) of Cu_2_S and CuS cluster are shown in Fig. 4. In addition, the Figures of Cu_3_S^+^ and Cu_5_S^−^ clusters are discussed and plotted in Figures S2 and S3 (see S1). From Fig. 4, the band gaps of Cu_2_S are larger than that of CuS, and the TDOS of Fermi level for Cu_2_S clusters is higher than CuS clusters, indicating the metallicity of CuS cluster is weakened due to a Cu ejection. We can also note that the TDOS of Cu_2_S clusters mainly comes from copper atoms in the region from −0.4 to −0.2 a.u. and sulfur atom in the region from 0.05 to 0.15 a.u. As evidenced by the diagram, the HOMO level is dominated primarily by the S-*p* orbital and partially by the Cu-*p*, *d* orbitals, in which Cu-*p* energy level is lower than Cu-*d* and S-*p* orbital energy level is much higher than Cu-*d*. The contribution from Cu-*s* and S-*s* orbital is almost zero. At LUMO case, the level is composed of Cu-*s*, *p*, *d* orbitals and S-*s*, *p* orbitals. The major contributions come from Cu-*s* orbital. Moreover, the orbital energy levels of Cu-*p* and S-*p* are higher than that of S-*s*; the contribution from Cu-*d* orbital is very small. These results provide a certification for our discussion based on molecular orbital composition analysis.

In order to decipher the chemical bonding of cluster, we performed a detailed analysis based on the localized orbitals resulting from the AdNDP method. AdNDP method, which is developed by Zubarev and Boldyrev^39^, is a very efficient and visual approach to the interpretation of the molecular orbital because it is an extension of the natural bond orbital analysis. It represents the molecular electronic structure in terms of *n*-center two-electron (*n*c-2e) bonds, the familiar lone pairs (1c-2e) and localized 2c-2e bonds or delocalized *n*c-2e bonds (3 ≤ n ≤ total number of atoms in the system). Moreover, the occupation numbers (ON) are expected to be close to the maximum values (ON = 2.00|e|).

**Figure 5:**
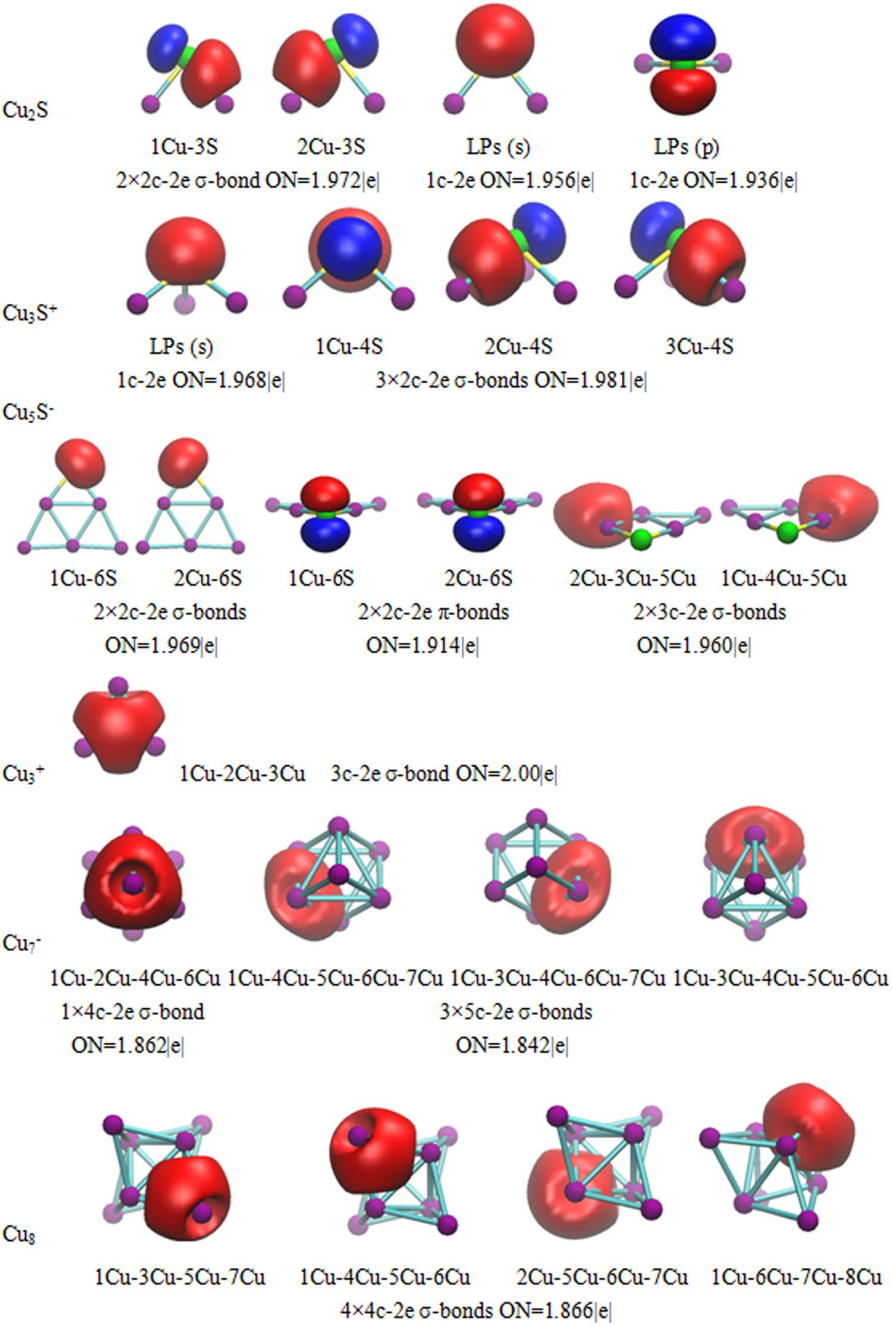
AdNDP chemical bonding analysis of the Cu_2_S, Cu_3_S^+^, Cu_5_S^−^, Cu_3_^+^, Cu_7_^−^ and Cu_8_ clusters. ON denotes occupation number.

When performing AdNDP approach to characterize the chemical bonding in Cu_2_S, the full-filled 3*d* orbital of Cu are not shown in this figure with ON ranging from 1.96 to 1.99 |e|. Excluding Cu (3*d*) orbital, Cu_2_S has 8 valence electrons with each copper atom contributing one valence electron and each sulfur atom contributing six valence electrons. The results of Cu_2_S cluster are depicted in Fig. 5. AdNDP analyses find four electrons are localized along the two localized Cu-S two-center two-electron (2c-2e) σ-bonds (ON = 1.972|e|). The remaining 4 electrons contain two lone pairs (LPs) with one *s*-type (ON = 1.956 |e|) and one *p*-type (ON = 1.936 |e|) on the sulfur atom. More specifically, per 2c-2e bond possesses the atomic contribution of 39.28%Cu + 60.72%S. In Cu-S 2c-2e bond, Cu-*s* and *p* offer 34.22% and 2.67% contribution to the Cu-based orbital, the S-based orbital possess the contributions of S-*p* (52.57%) and S-*s* (7.76%). Evidently, the Cu-S 2c-2e σ-bond is mainly dominated by the components from Cu-*s* and S-*p* type orbitals.

For Cu_3_S^+^ cluster, the 8 valence pairs are localized as one LPs with one S-*s* (ON = 1.968 |e|) and three localized Cu-S 2c-2e σ-bonds (ON = 1.981 |e|). The atomic contributions come from 32.61%Cu + 67.39%S for per 2c-2e bond. In Cu-S 2c-2e bond, the Cu-based orbital consist of Cu-*s* (27.10%) and Cu-*p* (3.96%); the corresponding S-based orbital is composed of 51.18% S-*p* and 16.22% S-*s*. Obviously, Cu-*s* and S-*p* possess three localized Cu-S 2c-2e bond. In addition, there is no covalent bonding between Cu and Cu atoms, indicating that interaction between Cu and Cu atoms is a kind of non-Lewis interaction.

There are 12 valence pairs in Cu_5_S^−^ cluster, analyses reveal two localized 2c-2e σ-bonds (ON = 1.969 |e|), two localized 2c-2e π-bonds (ON = 1.914 |e|), and two delocalized Cu-Cu-Cu 3c-2e σ-bonds (ON = 1.960 |e|). For the four localized 2c-2e bond, it attribute to the high symmetry structure and high atomization energy. And, each 2c-2e σ-bond involves the atomic contribution of 15.75%Cu + 84.25%S. For the bond, each Cu and S atoms can be considered as contributing Cu-*s*, -*p* (4.22%, 3.03%) and S-*s*, −*p* (74.14%, 9.9%), respectively. Furthermore, the atomic orbital composition from 2c-2e π-bond is obviously different from 2c-2e σ-bond. In 2c-2e π-bond, the Cu-S bond are consisted of 5.9%Cu + 94.1%S. Cu-based orbital is most from of 5.91% Cu-*p*. S-based orbital possess the atomic contribution of 94.08% S-*p*. In addition, there are two 3c-2e σ-bond exist in Cu_5_S^−^ cluster, each of atomic contribution involve 15.93%Cu(2) + 47.75%Cu(3) + 24.91%Cu(5). In Cu(2)-Cu(3)-Cu(5) 3c-2e bond, bridged Cu(2)-*s* and *p* contribute 11.19% and 4.02% to the Cu(2)-based orbital, Cu(3)-based orbital is completely consistent with 46.93% Cu-*s*, whereas Cu(5)-*s* and *p* contribute 20.67% and 3.34% to the Cu(5)-based orbital, respectively. Evidently, Cu(3)-*s* and Cu(5)-*s* orbitals appear to be the major contribution to the Cu(2)-Cu(3)-Cu(5) bridging bond in *C*_2v_ Cu_5_S^−^ cluster. For the Cu(1)-Cu(4)-Cu(5) 3c-2e bond, the atomic contribution are like as the results in Cu(2)-Cu(3)-Cu(5) 3c-2e bond.

For Cu_3_^+^ cluster, the results found that a pair of electrons is located inside of the copper triangle being delocalized over all three copper atoms. The occupation number of 3c-2e Cu-Cu-Cu σ-bonds is equal to the ideal value of 2.00 |e|. Due to the same contribution from the different three copper atoms, the corresponding atomic contribution is 33.33%Cu(1) + 33.33%Cu(2) + 33.33%Cu(3). Moreover, the 3c-2e Cu-Cu-Cu σ-bond possesses the orbital contribution of Cu-*s* (30.87%) and *p* (2.46%). The major contribution to the 3c-2e σ-bonds come from three Cu-*s* type orbital.

There exist 8 valence electrons in Cu_7_^−^ cluster. Analysis indicate that there are one delocalized 4c-2e σ-bond (ON = 1.862 |e|) in the trigonal pyramid structure and three delocalized 5c-2e σ-bonds (ON = 1.842 |e|) in three tetrahedral edges. For the 4c-2e Cu(1)-Cu(2)-Cu(4)-Cu(6) σ-bond, this bond can be viewed as contributing 17.54%Cu(1) + 33.22%Cu(2) + 17.55%Cu(4) + 17.55%Cu(6). Moreover, Cu(2)-*s* and *p* contribute 32.41% and 0.62% to the Cu(2)-based orbital; In Cu(1, 4, 6)-based orbital, each Cu atom possess the same contribution from Cu-*s* (13.78%) and Cu-*p* (3.33%). So, in 4c-2e Cu(1)-Cu(2)-Cu(4)-Cu(6) σ-bond, the major contribution is composed of Cu(1, 2, 4, 6)-*s* orbitals. For the 5c-2e σ-bond, the contribution from different copper atom is absolutely same. Cu(1)-Cu(4)-Cu(5)-Cu(6)-Cu(7) are chosen as representative examples. The corresponding atomic contribution is composed of 11.28%Cu(1) + 11.28%Cu(4) + 20.25%Cu(5) + 15.54%Cu(6) + 20.24%Cu(7). Moreover, Cu(1, 4)-*s*, -*p* offer 2.11% and 8.53% to the Cu(1, 4)-based orbital. Cu(5, 7)-*s* and *p* have 17.92% and 2.01% for the Cu(5, 7)-based orbital. At last, the contribution of Cu(6)-based orbital involves Cu-*s* (9.90%) and Cu-*p* (4.81%), respectively. In conclusion, in the 5c-2e σ-bond, Cu (5, 7, 6)-*s* provide the major contribution.

According to the AdNDP analysis of the bare Cu_8_ cluster, 8 valence electrons possess four delocalized 4c-2e σ-bonds (ON = 1.866 |e|). The corresponding atomic contribution from Cu(1)-Cu(2)-Cu(4)-Cu(6) is consistent with 17.54%Cu(1) + 33.22%Cu(6) + 17.55%Cu(7) + 17.54% Cu(8). Furthermore, Cu(1, 6, 7)-*s*, -*p* provide 11.91% and 2.89% to the Cu(1, 6, 7)-based orbital. Cu(8) -*s*, -*p* possess 30.23% and 0.84% for the Cu(8)-based orbital. Obviously, Cu-*s* provides the major contribution to four delocalized 4c-2e σ-bonds in Cu_8_ cluster.

**Figure 6:**
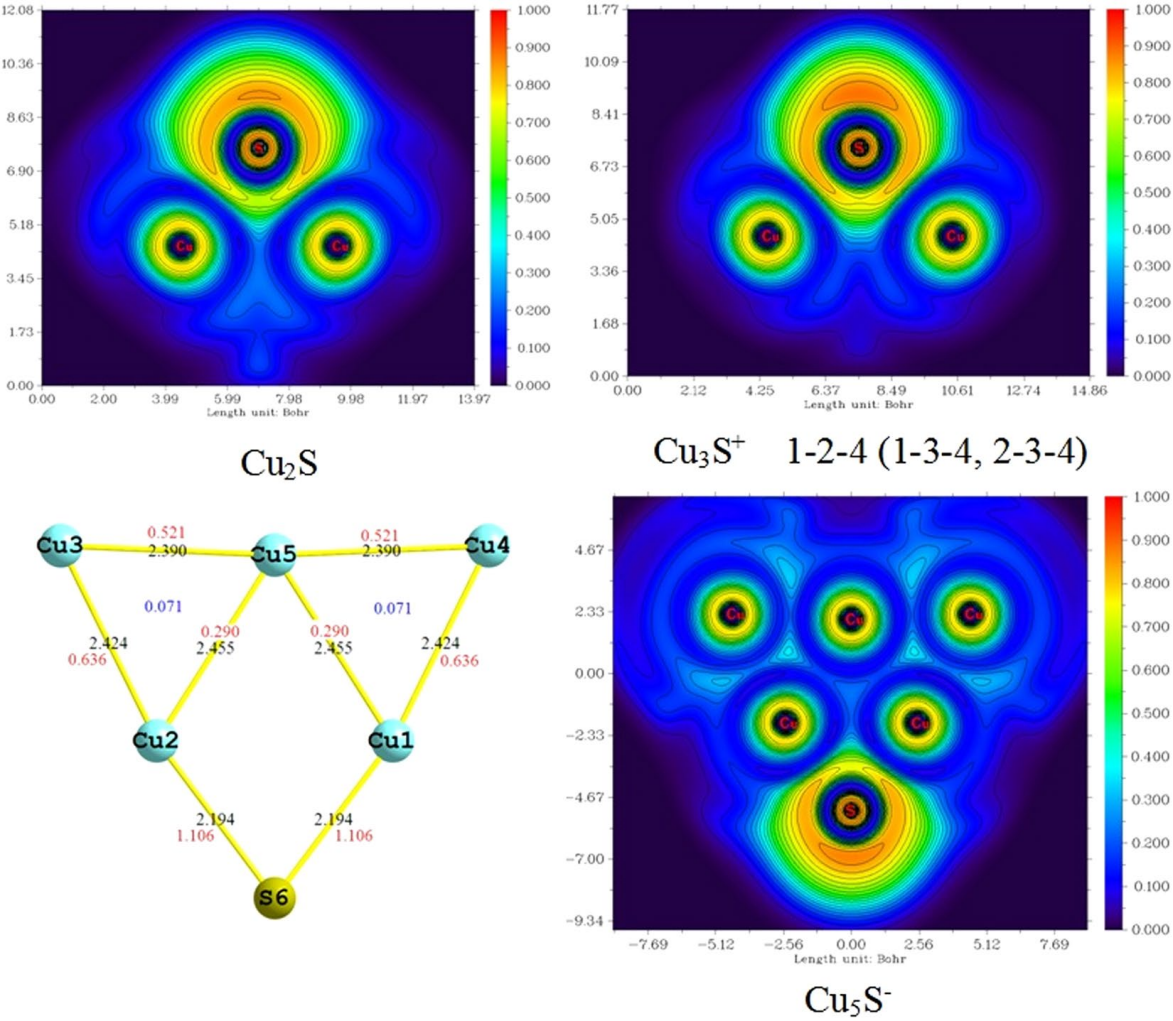
Geometrical parameters (bond length, in Å) of Cu_2_S, Cu_3_S^+^ and Cu_5_S^−^ clusters and their ELF cut planes and Mayer Bond order.

In order to study the bond interactions of doped systems, we conducted the electron localization function (ELF) analysis using the Multiwfn program package^40^. The ELF is usually interpreted as the probability of finding an electron pair localized in some region of the real space. There are three special cases: ELF = 0 indicates that there is no electron density between atomic orbitals; ELF = 0.5 shows the regions with bonding of a metallic character; ELF = 1 corresponds to perfect localization, and means covalent bonds and inner shell or lone pair electrons. To elucidate the type of bonding in clusters, bond length and Mayer bond order and ELF cut planes for Cu_2_S, Cu_3_S^+^ and Cu_5_S^−^ cluster are illustrated in Fig. 6. We can see that the ELF values near Cu atoms are very high which associated with 1s core electrons, whereas the ELF values of regions between copper atoms are very small (nearly zero), it means that the chemical bonding between Cu and Cu atoms can be neglected. The ELF values of bonds are small between Cu and S atoms, indicating that there exist typical of ionic bonding in Cu_2_S, Cu_3_S^+^ and Cu_5_S^−^ clusters. In addition, the distribution of ELF value near the S atom is not spherically symmetric, and there is a region with higher localized degree. This implies that the S atom through *sp* hybridization from normal two-center σ bonds with the near copper atoms. The present results are in good agreement with AdNDP chemical bonding analysis. Moreover, in Cu_5_S^−^ cluster, it is clear that electrons are mainly localized within the two outside triangles, corresponding to the three-center two-electron bond (3c-2e). Simultaneously, Mayer three-center bond order analyses show that the value of outside triangle is 0.071e. In addition, based on the two-center bond order analysis, the bond order of boundary Cu-Cu bond (0.521) is markedly larger than the inner one (0.290). Results indicate that the bond of boundary Cu-Cu is more stable than the inner ones; the bond of boundary Cu-S is more stable than that of Cu-Cu. At last, comparing the different bond lengths, we can find that short bond lengths lead to strong interatomic interactions, which play an important role in stabilizing structures.

## Conclusions

In this paper, we have performed a global minimum search for the lowest energy structures of neutral, cationic and anionic Cu_n+1_ and Cu_n_S (n = 1–12) clusters by using CALYPSO method in combination with density functional theory. The results are summarized as below:

(i) The structure searches show that the lowest energy structures are sensitive to the charge states. A new global minimum state of Cu_9_ cluster is obtained, which is more stable than previous work. For Cu_n_S^+/0/−^ clusters, Cu directly added on the Cu_n−1_S clusters to form the Cu_n_S clusters is the dominant growth pattern.
(ii) Sulfur atom doping has considerable influence on not only geometries and properties but also the valence electron count of copper clusters. Trends of the atomic binding energies, second-order difference of energies and HOMO-LUMO gaps showed that the clusters containing even number of electrons maintain greater stability than odd number of electrons. In addition, Cu_3_^+^, Cu_8_, Cu_7_^−^, Cu_2_S and Cu_3_S^+^ follow the trends predicted by the Jellium model with the 2, 8 valence electron systems being the most stable. However, Cu_5_S^−^ cluster with 12 valence electrons does not correspond to the magic numbers also exhibit an increased stability.
(iii) The calculated HOMO-LUMO gaps range from 1.01–4.29 eV, 0.8–2.96 eV, 1.26–2.53 eV, respectively, which make Cu_n_S^+/0/−^ clusters suitable candidates for renewable energy sources (especially Cu_n_S^+^ clusters). Density of states reveals that the orbital contribution of HOMO level is dominated by the S-*p* and Cu-*s*, *p*, *d*, the orbital contribution from S-*s* is almost zero. In LUMO level, the orbital contributions are composed of Cu-*s*, *p*, *d* and S-*s*, *p*, respectively. Moreover, the structural symmetry effect corresponding orbital composition.
(iv) AdNDP analyses show the chemical bonding patterns are in good agreement with the geometric structures and the ELF and Mayer Bond order analysis. The bond of boundary Cu-Cu is more stable than the inner ones. The bond of boundary Cu-S is more stable than that of Cu-Cu.

## Methods

The lowest energy structures of neutral and charged Cu_n+1_ and Cu_n_S clusters are searched by the swarm-intelligence based CALYPSO structure prediction method^41–43^. This method is based on globally minimizing potential energy surfaces, merging *ab initio* total energy calculations with CALYPSO cluster prediction through particle swarm optimization. It has been successful in correctly predicting structures for various systems^44–46^. In this prediction method, each generation contain 20 structures, 70% of which are generated by particle swarm optimization (PSO). PSO is a population approach based stochastic optimization technique developed by Eberhart and Kennedy in 1995^47, 48^. The others are new and will be generated randomly. In process of searching, a sequence of 50 generations of structural candidates is followed to achieve convergence. So, we can achieve 1000 structurally different low-lying isomers. Among the 1000 isomers, the top fifty low-lying isomers are collected as candidates. Those structures with energy difference from the lowest energy isomers less than 0.3 eV are further optimized to identify the lowest energy structure. The further geometry optimizations are performed with no symmetry constraints at the level of the generalized gradient approximation (GGA) using the exchange-correlation functional B3P86^49, 50^ as implemented in the GAUSSIAN09 package^51^. The basis set labeled GENECP, i. e. the LanL2DZ basis set for Cu atom and 6-311 + G* basis set for S atom, respectively^52, 53^.

## Electronic supplementary material

Supplementary Information
