# Supplementary material for: Insights into the structures and electronic properties of Cun+1μ and CunSμ (n = 1–12; μ = 0, ±1) clusters
Source: Sci Rep. 2017 May 2;7:1345. doi: 10.1038/s41598-017-01444-6 (PMC5430952; doi:10.1038/s41598-017-01444-6)
Supplement: Supplementary file 1 — Supplementary Information [file 41598_2017_1444_MOESM1_ESM.doc]

**Insights into the structures and electronic properties of Cu_n+1_*^μ^*and Cu_n_S*^μ^* (n=1-12; *μ*=0, ±1) clusters**

Cheng-Gang Li ^a, b^, Zi-Gang Shen ^a^, Yan-Fei Hu c [[1]](#footnote-2)*, Ya-Nan Tang ^a^, Wei-Guang Chen ^a^, Bao-Zeng Ren ^b^

*^a^* *College of Physics and Electronic Engineering, Quantum Materials Research Center, Zhengzhou Normal University, Zhengzhou 450044, China*

*^b^ School of Chemical Engineering and Energy, Zhengzhou University, Zhengzhou 450001, China*

*^c^ School of Science, Sichuan University of Science ＆ Engineering, Zigong 643000, China*

^*^Correspondence to: Yan-Fei Hu, School of Science, Sichuan University of Science ＆ Engineering, Zigong 643000, China. E-mail address: [yanfei_­­hu1982@suse.edu.cn](mailto:huyanfei1982@126.com)

**Table S1.** Electronic states, symmetries and frequency of the lowest-energy of Cu_n_*^μ^*and Cu_n_S*^μ^* (n=1-12; *μ*=0, ±1) clusters.

|  | Cu_n_ | | | Cu_n_^+^ | | | Cu_n_^-^ | | |
| --- | --- | --- | --- | --- | --- | --- | --- | --- | --- |
| *n* | sta | sym | Frequency | sta | sym | Frequency | sta | sym | Frequency |
| 2 | ^1^Σ | *D*_∞h_ | 267 | ^2^Σ | *D*_∞h_ | 194 | ^2^Σ | *D*_∞h_ | 197 |
| 3 | ^2^B_2_ | *C*_2V_ | 82,162,248 | ^1^A_1_ | *D*_3h_ | 165,165,250 | ^1^Σ | *D*_∞h_ | 42,2, 243 |
| 4 | ^1^A_g_ | *D*_2h_ | 59,115,124  153,227,262 | ^2^B_1_ | *D*_2h_ | 31,110,113  134,200,239 | ^2^B_2_ | *D*_2h_ | 50,109,128  157,215,216 |
| 5 | ^2^A_1_ | *C*_2V_ | 40,41,101  113,138,164  201,217,257 | ^1^A_1_ | *D*_2d_ | 18,41,41  108,143,143  196,237,268 | ^1^A_1_ | *C*_2V_ | 39,51,87  111,124,138  179,212,254 |
| 6 | ^1^A_1_ | *D*_3h_ | 30,37,37  100,100,121  121,168,193  197,259,259 | ^1^A_2_ | *C*_2V_ | 47,68,69  101,108,109  124,131,166  181,217,246 | ^2^B_1_ | *C*_2V_ | 46,63,95  98,124,126  131,144,162  193,219,228 |
| 7 | ^2^A_2_ | *D*_5h_ | 61,61,109  109,120,126  126,132,151  151,161,161  215,215,230 | ^1^A_1_ | *D*_5h_ | 77,77,87  87,113,113  117,123,123  138,138,148  213,213,245 | ^1^A_1_ | *C*_3v_ | 62,62,92  92,105,117  124,124,140  169,169,200  200,212,232 |
| 8 | ^1^A_1_ | *T*_d_ | 49,49,94  94,94,127  127,127,137  137,137,147  170,170,221  221,221,224 | ^2^A’ | *C*_S_ | 52,66,74  90,102,112  113,122,124  128,133,136  142,159,187  203,220,239 | ^2^A_1_ | *D*_2d_ | 60,79,79  79,93,98  98,106,121  151,153,153  176,184,198  198,211,219 |
| 9 | ^2^A_2_ | *C*_2v_ | 57,63,72  82,93,98  106,107,115  115,117,121  131,137,161  170,182,190  211,217,236 | ^1^A_1_ | *C*_2V_ | 46,66,68  71,100,100  110,113,114  121,123,127  135,150,157  158,177,198  218,233,251 | ^1^A_1_ | *C*_2v_ | 49,61,78  79,92,92  97,101,115  115,127,140  149,159,175  178,200,202  210,216,228 |
| 10 | ^1^A_1_ | *D*_2d_ | 55,62,62  80,86,86  111,114,114  117,123,131  131,131,145  145,159,159  171,207,220  220,220,225 | ^2^A_1_ | *D*_2d_ | 50,69,69  78,78,88  104,104,111  113,118,120  124,124,139  139,154,154  154,208,208  209,217,228 | ^2^A’ | *C_s_* | 38,58,63  75,76,88  96,97,108  109,111,124  133,134,148  154,164,164  179,196,209  212,218,235 |
| 11 | ^2^B | *C*_2_ | 57,58,61  75,82,97  98,103,105  105,106,114  115,124,130  134,137,156  159,168,170  185,205,205  210,229,230 | ^1^A’_1_ | *D_3h_* | 41,41,68  68,82,82  96,96,103  106,106,119  119,122,127  127,149,172  172,173,176  176,203,204  204,231,239 | ^1^A_1_ | *C_2v_* | 44,45,64  66,74,82  85,91,91  99,113,116  119,121,129  142,148,155  166,178,189  192,203,203  213,223,226 |
| 12 | ^1^A’ | *C*_s_ | 49,59,60  75,79,81  91,98,98  99,105,108  118,121,122  131,134,138  140,142,160  165,174,176  195,207,211  229,230,231 | ^2^A | *C*_1_ | 53,55,67  69,79,81  83,88,94  99,105,108  111,116,123  129,130,132  137,138,148  156,163,172  190,200,202  220,226,246 | ^2^A’ | *C*_s_ | 60,62,66  75,76,88  91,93,93  101,104,104  108,114,117  127,129,132  138,146,152  163,172,178  202,202,208  214,231,234 |
| 13 | ^2^A | *C*_2_ | 35,54,60  61,66,70  74,79,82  95,97,104  109,111,117  123,129,131  138,140,149  153,164,169  183,183,196  198,206,216  223,224,237 | ^1^A’ | *C*_s_ | 51,53,65  66,72,77  87,89,94  95,97,99  105,113,115  117,123,127  134,139,143  143,145,148  169,170,172  199,204,220  231,234,239 | ^1^A_1_ | *C*_2v_ | 32,43,50  54,61,69  78,81,88  98,100,100  104,108,123  123,124,133  139,144,150  156,157,178  180,189,193  199,206,222  225,227,252 |
|  | Cu_n_S | | | Cu_n_S^+^ | | | Cu_n_S^-^ | | |
| *n* | sta | sym | Frequency | sta | sym | Frequency | sta | sym | Frequency |
| 1 | ^2^Σ | *C*_∞_*_v_* | 381 | ^2^Σ | *C*_∞_*_v_* | 358 | ^1^Σ | *C*_∞_*_v_* | 402 |
| 2 | ^1^A’ | *C*_2V_ | 85,332,408 | ^2^B_1_ | *C*_2V_ | 61,319,358 | ^2^A’ | *C*_2v_ | 147,231,363 |
| 3 | ^2^A_1_ | *C*_3V_ | 89,89,146  219,219,369 | ^1^A_1_ | *C*_3V_ | 46,46,66  345,346,346 | ^1^A_1_ | *C*_2v_ | 70,111,157  217,236,366 |
| 4 | ^1^A_1_ | *C*_3V_ | 85,85,120  120,147,204  217,217,371 | ^2^A_1_ | *C*_3V_ | 63,63,89  93,93,168  267,267,368 | ^2^A’ | *C*_s_ | 42,70,99  121,143,197  203,276,371 |
| 5 | ^2^A’ | *C*_s_ | 54,62,82  99,102,130  161,191,206  213,248,362 | ^1^A_1_ | *C*_3V_ | 44,44,92  92,116,116  116,190,207  207,213,427 | ^1^A_1_ | *C*_2v_ | 37,57,76  87,118,125  144,179,194  240,302,379 |
| 6 | ^1^A’ | *C*_s_ | 34,69,78  81,94,101  112,138,169  177,203,214  243,281,370 | ^2^B_2_ | *C*_2V_ | 74,75,87  87,110,110  121,127,134  165,173,175  202,251,332 | ^2^A’ | *C*_s_ | 35,72,76  104,115,124  127,136,153  153,168,213  213,242,344 |
| 7 | ^2^A’’ | *C*_s_ | 61,74,78  89,102,103  117,123,131  150,157,166  176,210,213  226,264,369 | ^1^A’ | *C*_s_ | 31,31,59  70,75,87  96,109,114  127,135,162  181,202,239  282,314,377 | ^1^A’ | *C*_s_ | 38,73,80  99,103,105  118,123,145  147,156,165  181,195,210  223,231,351 |
| 8 | ^1^A’ | *C*_s_ | 62,65,73  75,79,95  105,112,120  121,141,143  158,164,172  190,216,236  255,274,374 | ^2^A’ | *C*_s_ | 51,65,77  80,82,83  96,110,120  123,140,147  149,154,160  188,206,232  271,286,373 | ^2^A’ | *C*_s_ | 61,65,82  83,91,102  104,104,113  131,141,144  158,165,178  190,196,209  219,227,346 |
| 9 | ^2^A’ | *C*_s_ | 34,42,75  77,80,82  100,102,103  121,122,133  137,148,153  158,160,194  196,224,235  260,269,367 | ^1^A_1_ | *C*_3V_ | 62,62,76  84,84,86  86,88,116  116,119,131  131,152,153  153,173,176  195,235,235  287,287,381 | ^1^A’ | *C*_s_ | 27,47,67  68,82,91  96,97,109  114,126,127  135,148,151  157,171,190  205,218,230  236,260,354 |
| 10 | ^1^A | *C*_1_ | 53,58,67  76,76,93  96,97,101  108,112,117  118,124,137  146,151,152  163,172,185  203,226,230  246,260,369 | ^2^A’ | *C*_s_ | 43,58,70  79,80,82  88,92,95  104,108,110  113,126,128  134,150,171  176,178,180  200,213,234  235,267,324 | ^2^A | *C*_1_ | 52,56,62  69,71,87  93,94,98  104,112,118  123,129,138  147,158,160  169,174,192  201,213,219  229,247,353 |
| 11 | ^2^A’’ | *C*_s_ | 49,58,66  70,76,80  88,94,98  106,107,110  114,125,128  133,140,146  147,155,155  167,190,190  198,224,250  250,272,366 | ^1^A’ | *C*_s_ | 17,29,55  61,69,77  80,85,92  97,99,105  111,119,120  121,143,144  146,155,175  181,191,205  210,234,242  264,294,378 | ^1^A’ | *C*_s_ | 43,47,56  56,71,79  82,90,92  100,102,107  113,118,124  133,143,146  159,162,176  176,194,203  210,210,229  229,275,362 |
| 12 | ^1^A_1_ | *C*_2V_ | 65,68,71  71,73,83  85,86,99  102,103,107  110,114,115  117,120,120  139,150,154  160,167,171  173,198,210  219,238,241  253,262,339 | ^2^A | *C*_1_ | 38,41,51  65,70,74  82,85,86  92,99,104  106,113,119  121,128,134  140,148,153  162,168,179  185,190,194  218,231,236  254,286,337 | ^2^A | *C*_1_ | 48,54,66  68,76,79  83,86,90  96,100,103  107,113,118  123,127,134  139,150,157  157,168,175  184,194,200  202,216,219  234,268,329 |

**Figure S1.** The calculated and experimental static mean polarizablity <α>, vertical ionization potentials (VIP) and electron affinities (VEA) of the lowest energy structures for neutral and charged Cu_n+1_ (n=1-12) clusters.

**Figure S2.** Total densities of states for Cu_2_S^+^ cluster (a). Total and partial densities of states of Cu_3_S^+^ clusters (b). Partial densities of states for copper atom of Cu_3_S^+^ clusters (c). (Full width at half maximum (FWHM) =0.02 a.u.) The dashed line indicates the HOMO and LUMO energy.

**Figure S3.** Total densities of states for Cu_3_S^-^ cluster (a). Partial densities of states of Cu_5_S^-^ clusters (b). Partial densities of states for copper atom of Cu_5_S^-^ clusters (c). (Full width at half maximum (FWHM)=0.02 a.u.) The dashed line indicates the HOMO and LUMO energy.

1. ^*^ Correspondence to: Yan-Fei Hu, School of Science, Sichuan University of Science ＆ Engineering, Zigong 643000, China. E-mail address: [yanfei_­­hu1982@suse.edu.cn](mailto:huyanfei1982@126.com) [↑](#footnote-ref-2)
